# Impact and Cost-Effectiveness of a Comprehensive *Schistosomiasis japonica* Control Program in the Poyang Lake Region of China

**DOI:** 10.3390/ijerph10126409

**Published:** 2013-11-28

**Authors:** Qing Yu, Geng-Ming Zhao, Xian-Lin Hong, Eric A. Lutz, Jia-Gang Guo

**Affiliations:** 1 Department of Schistosomiasis, National Institute of Parasitic Diseases, Chinese Center for Disease Control and Prevention, 207 Rui Jin Er Road, Shanghai 200025, China; E-Mail: yuqing1@chinacdc.cn; 2 World Health Organization Collaborating Center for Malaria, Schistosomiasis, and Filariasis—Key Laboratory of Parasite and Vector Biology, Ministry of Health, 207 Rui Jin Er Road, Shanghai 200025, China; 3 Department of Epidemiology, School of Public Health, Fudan University, 130 Dong An Road, Shanghai 200032, China; E-Mail: gmzhao@shmu.edu.cn; 4 Jingxian Schistosomiasis Control Station, JingXian 331700, China; E-Mail: xuefangzhan2004@yahoo.com.cn; 5 Environmental and Occupational Health Section, Division of Community, Environment, and Policy, Mel and Enid Zuckerman College of Public Health, University of Arizona, 1656 E. Mabel Street, Room 113, Tucson, AZ 85724, USA; 6 World Health Organization 20, Avenue Appia, CH-1211 Geneva 27, Switzerland

**Keywords:** *Schistosomiasis japonica*, comprehensive control program, cost-effectiveness, Poyang Lake region

## Abstract

*Schistosomiasis japonica* remains a significant public-health problem in China. This study evaluated cost-effectiveness of a comprehensive schistosomiasis control program (2003–2006). The comprehensive control program was implemented in Zhangjia and Jianwu (cases); while standard interventions continued in Koutou and Xiajia (controls). Incurred costs were documented and the schistosomiasis comprehensive impact index (SCI) and cost-effectiveness ratio (Comprehensive Control Program Cost/SCI) were applied. In 2003, prevalence of *Schistosoma japonicum* infection was 11.3% (Zhangjia), 6.7% (Jianwu), 6.5% (Koutou), and 8.0% (Xiajia). In 2006, the comprehensive control program in Zhangjia and Jianwu reduced infection to 1.6% and 0.6%, respectively; while Koutou and Xiajia had a schistosomiasis prevalence of 3.2% and 13.0%, respectively. The year-by-year SCIs in Zhangjia were 0.28, 105.25, and 47.58, with an overall increase in cost-effectiveness ratio of 374.9%–544.8%. The SCIs in Jianwu were 16.21, 52.95, and 149.58, with increase in cost-effectiveness of 226.7%–1,149.4%. Investment in Koutou and Xiajia remained static (US$10,000 unit cost). The comprehensive control program implemented in the two case villages reduced median prevalence of schistosomiasis 8.5-fold. Further, the cost effectiveness ratio demonstrated that the comprehensive control program was 170% (Zhangjia) and 922.7% (Jianwu) more cost-effective. This work clearly shows the improvements in both cost and disease prevention effectiveness that a comprehensive control program-approach has on schistosomiasis infection prevalence.

## 1. Introduction

Over the past sixty years, *Schistosomiasis japonicum* control efforts have resulted in significant disease reduction across China, as evidenced by the Report of Schistosomiasis Status in China which estimated 412,927, 365,770, 325,825, and 286,386 cases per year for 2008–2011, respectively. However, schistosomiasis remains a significant and costly public health issue along the lower reaches of the Yangtze River Basin and in some mountainous regions of Yunnan and Sichuan Province. Additional provinces where schistosomiasis remains endemic include Hunan, Hubei, Anhui, Jiangxi and Jiangsu. Many villages surrounding Poyang Lake, China’s largest freshwater lake are endemic with schistosomiasis, despite extensive, focused control efforts. There are nine counties within Jiangxi Province that remain endemic for the parasite, with an estimated 76,652 people infected (2011) [1,2,3,4].

Prior to the 1980s, the major intervention measures focused on large-scale environmental control using molluscicides to eliminate or reduce snail populations. When the drug praziquantel was introduced in the 1980s, schistosomiasis mitigation efforts shifted to chemotherapy-based morbidity control, supplemented with local snail reduction using niclosamide. Applying this control methodology resulted in reductions of overall prevalence of schistosomiasis infection [5,6,7,8]; since the early twenty-first century, many “comprehensive” control programs have been implemented on a trial basis in endemic areas of China, while in other areas the standard chemotherapy/snail reduction programs continue, even today. Both of these programs have claimed success [9,10]. However, Chen H. *et. al*., reported that around Poyang Lake in 2002, human infection rates had rebounded in several villages, including: Songfeng Village, Yongxiu County (36.67% infection), Yufeng Village (33.08%), Nanchang County, Nanji Village (13.67%), Xinjian County [11,12]. Control of schistosomiasis is complicated by ease of re-infection and Wang *et al*., has noted that under the current approach the interruption of transmission seems unsustainable [13]. Specifically, Wang suggests controlling infectious sources using a multi-component, “comprehensive” control strategy (Comprehensive Program) that simultaneously includes: (1) grazing and marshland isolation, (2) replacing bovines with tractors, (3) environmental management to suppress snail populations, (4) re-purposing agricultural resources, (5) improving access to water and sanitation facilities, (6) synchronous chemotherapy of human and livestock, and (7) health education. 

Historically during control efforts, administration of health surveys and collection of metrics, including annual infection rates in both humans and bovines, infection rate of snails, and density of infected snails have been common. Further, cost information related to intervention expenses from personal salary, per diem and transportation, costs associated with human screening and treatment, snail surveys and control, and health education are also typically archived. However, making use of this available data, in aggregate, is uncommon. Currently, very little objective data pertaining to the comparative impact and cost-effectiveness of schistosomiasis control programs exists. Therefore, the current study evaluates the comparative cost-effectiveness, and impact using a schistosomiasis comprehensive impact index (SCIs), of each program type over a four-year period, in four comparable lower Yangtze River Basin villages in China. 

## 2. Experimental Section

**Figure 1:**
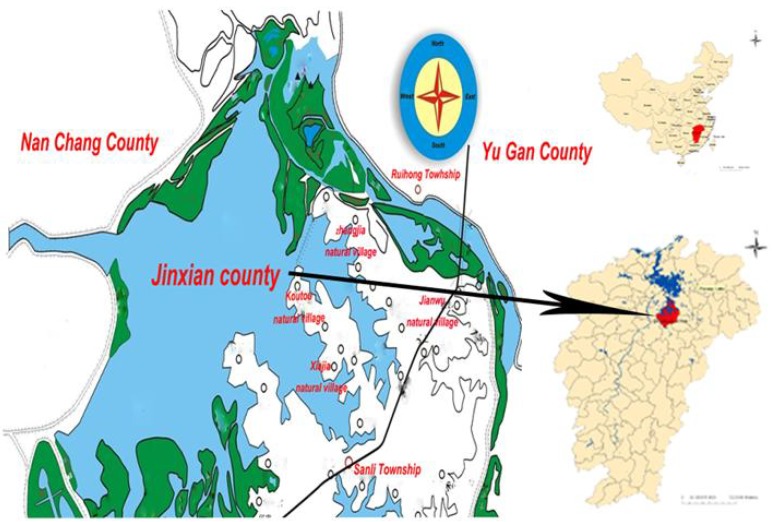
The geographical distribution of the cases villages (Zhangjia and Jianwu villages) and the control villages (Koutou and Xiajia village) in Sanli Township of Jinxian County, Jiangxi Province in China.

A four-year, retrospective, case-control study was conducted using data from four villages located in Sanli Township of Jingxian County, Jiangxi Province, China (Figure 1). The two “case” villages where the comprehensive schistosomiasis control intervention (Comprehensive Program) was applied were Zhangjia and Jianwu. The two “control” villages, Koutou and Xiajia, received the standard control intervention (Standard) throughout the study period. Identical Standard control was applied in all four villages in Year 1 of the study and baseline surveillance was measured. In Years 2 and 3, the Comprehensive Program intervention was implemented in Zhangjia and Jianwu, while the Standard control measures continued in Koutou and Xiajia. In Year 4, all four villages received the Standard intervention. Basic endemic information for the four villages is summarized in Table 1. 

The Comprehensive Program implemented in Zhangjia and Jianwu included: (1) removing snail-containing pastures from agricultural use, (2) isolating snail contaminated marshland through community notification and removal of areas for human/livestock use, (3) replacing traditionally used bovines with tractors, (4) eliminating snails using niclosamide, (5) re-purposing specific agricultural lands, (6) improving sanitation and water supply infrastructure, (7) administering synchronous chemotherapy to both humans and livestock, and (8) conducting health education campaigns. In Koutou and Xiajia, the standard schistosomiasis control measures included synchronous population/animal chemotherapy and simultaneous environmental snail reduction using molluscicide, supplemented with population health education. The age of village populations sampled ranged from 6–60 years old.

**Table 1:** Baseline information for the four villages.

	Villages	Total population (person)	Total households	Average Income (US $/year)	Infection rate population (% prevalence)	Total buffalo (heads)	Infection rate of buffalo (%)	Area snails habitant (ha)	Area infection snail (ha)
Cases villages	Zhangjia	920	215	479.41	11.3	184	4.0	62.53	62.53
	Jianwu	1,226	286	478.09	6.7	393	4.0	1,031.00	1,031.00
Control villages	Koutou	698	162	477.94	6.5	85	4.5	39.23	25.40
	Xiajia	1,167	290	426.47	8.0	120	6.0	14.26	14.25

Using available 2003–2006 data from the four previously named villages, a retrospective impact and economic evaluation was performed. A schistosomiasis comprehensive impact index (SCI) was developed (Equation (1). Schistosomiasis Comprehensive Impact Index (SCI).) and intervention program costs were calculated (Equation (2). Control Program Costs.) to evaluate the cost/benefit of the respective control programs. Data included values for total number of humans and bovines infected annually and number of cases treated using chemotherapy delivery per year, snail distribution and density, total area (ha =10,000 m^2^) of snail habitats subjected to molluscicide, and total area (ha) of snail habitat elimination using niclosamide (as recommended by the WHO). Values related to chemotherapy treatment measures were used as effectiveness indicators. Cost information related to intervention expenses from personal salary, per diem and transportation, costs associated with human screening and treatment, snail surveys and control, and health education, were used to arrive at the cost-effectiveness of each program.

Cost-effectiveness was estimated using four indices: (1) unit cost of reducing human *S. japonicum* infection by 1%, (2) unit cost of reducing bovine infection by 1%, (3) ratio of cost-index for control program implementation (Comprehensive Program Costs/SCI), and (4) the schistosomiasis comprehensive impact index (SCIs) [14,15], as defined by:

SCI = 0.6 (0.4 PIRD + 0.25 EPGD + 0.35 UCIRD_10_) + 0.3 (0.4 LSDD + PSDD × 0.6) + 1.00 LIRD (1)

SCI—Schistosomiasis control comprehensive impact index
PIRD—decline in human infection rate
EPGD—decline in EPG (EPG, schistosome egg per gram)
UCIRD_10_—decline in infection rate of children aged <10 years old
LSDD—decline in density of living snails
PSDD—decline in density of infected snails
LIRD—decline in infection rate of livestock

The total cost of control activities was aggregated for each case village using a cost effectiveness ratio, described in Equation (2), as follows:

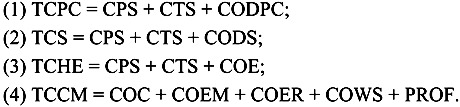
(2)

**All values in total cost:**

TCPC—screening and treatment for humans and bovines
CPS—professional staff
CTS—temporary staff
CODPC—equipment and drugs for population and cattle
TCS—surveys and mollusciciding snails
CODS—equipment and drugs for snails
TCHE—health education
COE—equipment, including facilities and health promotion materials
TCCM—comprehensive measures
COC—grazing and marshland constraints and replacing bovines with tractors
COEM—snail control through environmental management (e.g. tree planting)
COER—land re-purposing agriculture development
COWS—water supply and sanitation improvement
PROF—staff (incl., *per diem*, travel, human/animal exams and treatment)

Statistical analyses were performed using SPSS software version 18 (SPSS Institute, Chicago, IL, USA). Chi-square tests (χ^2^) were used to test for differences between groups, with the alpha error threshold for significance set at *p* < 0.05.

## 3. Results and Discussion

### 3.1. S. japonicum Infections in Humans

Prior to the implementation of the Comprehensive Program in Zhangjia and Jianwu, a baseline survey was performed in all four study villages in Year 1 of the study. The prevalence of human *S. japonicum* infection was 11.3% in Zhangjia, 6.7% in Jianwu, 6.5% in Koutou, and 8.0% in Xiajia. This baseline found that no significant differences existed in the number of human infections across the “case” villages and “control” villages (χ^2^ = 0.996 *p* = 0.326). Mid-study post-intervention evaluations at the beginning of Year 2 indicated statistically significant reductions in human infections in the case villages of Zhangjia (¬86.3%) and Jianwu (­91.6%), over the control villages of Koutou (¬51.2%) and Xiajia (+38.6%, *p* < 0.05), as noted in Table 2.

At the end of Year 2, Year 3, and Year 4, in Zhangjia, human infection rates decreased from Year 1 baseline by 29.4%, 56.6%, and 55.3%, respectively. During the same time period in Jianwu, the schistosomiasis infection rates also fell by 50.1%, 48.9%, and 67.2%. In summary, three years following implementation of the Comprehensive Program in Zhangjia and Jianwu, human *S. japonicum* infection was reduced to 1.6% and 0.6%, respectively. In the control village of Koutou, end of Year 2, Yea 3, and Year 4, human infection rates decreased by 7.7%, 38.5%, and 20.8%. However, in Xiajia, human infection rates decreased in Year 2 from baseline by 27.8%, but increased in Year 3 and Year 4 by +2.5% and +119.9%. Koutou and Xiajia, where Standard schistosomiasis control measures continued throughout the study period, were observed to have schistosomiasis prevalence at the end of the same three years of 3.2% and 13.0%, respectively.

**Table 2:** The results of fecal examination on the population from first year to fourth year.

		No. of persons examined				No. with infection				Infection rate (% prevalence)			
	Village	Year 1	Year 2	Year 3	Year 4	Year 1	Year 2	Year 3	Year 4	Year 1	Year 2	Year 3	Year 4
Case	Zhangjia	300	300	519	580	34	24	18	9	11.3	8.0	3.5	1.6
	Jianwu	300	300	353	359	20	10	6	2	6.7	3.3	1.7	0.6
Control	Koutou	200	200	450	378	13	12	18	12	6.5	6.0	4.0	3.2
	Xiajia	200	182	405	315	16	15	24	41	8.0	8.2	5.9	13.0

### 3.2. S. japonicum Infections in Livestock

The baseline Year 1 surveys indicated that there were no significant differences in the number of bovine *S. japonicum* infections between the Zhangjia (case) and Koutou (control) (χ^2 ^= 0.2997, *p* = 0.686), nor Jianwu (case) and Xiajia (control) (χ^2 ^= 0.3728, *p* = 0.621). As the Comprehensive Program implemented in case villages included replacement of working bovines with tractors, there were no bovines in either case village until Year 4. In control villages, the number of bovine *S. japonicum* infections increased in Koutou from 6.0% in Year 1 to 7.1% in Year 4, but decreased in Xiajia from 6.7% to 6.0% in the same period. Table 3 provides a summary of the per village annual bovine schistosomiasis data. 

**Table 3:** The results of fecal examination on the livestock (water buffalo).

		No. of cattle examined				No. with infection				Infection rate (% prevalence)			
	Village	Year 1	Year 2	Year 3	Year 4	Year 1	Year 2	Year 3	Year 4	Year 1	Year 2	Year 3	Year 4
Case	Zhangjia	100	100	55	0	4	4	0	-	4.0	4.0	0.0	-
	Jianwu	100	100	115	0	4	4	1	-	4.0	4.0	0.9	-
Control	Koutou	50	66	60	28	3	3	4	2	6.0	4.5	6.7	7.1
	Xiajia	30	50	40	67	2	3	3	4	6.7	6.0	7.5	6.0

### 3.3. Snail Survey

Snail survey results indicated a reduction in the density of infected snails in case villages from 0.0048 snails/0.1 m^2^ (Zhangjia) and 0.0032 snails/0.1 m^2^ (Jianwu) in Year 1 to 0.0010 snails/0.1 m^2^ and 0 snails/0.1 m^2^ in Year 4, respectively. Simultaneously in control villages, the density of infected snails increased from 0.0016 snails/0.1 m^2^ and 0.0027 snails/0.1 m^2^ in Year 1 to 0.0049 snails/0.1 m^2^ and 0.0087 snails/0.1 m^2^ in Year 4 for Koutou and Xiajia, respectively. The aggregated Year 1 to Year 4 difference between the case and control counts of infected snails was significant (χ^2^ = 4.313, *p* = 0.038), with reduction in Zhangjia from 3.4% in the first year to 0.7% in the fourth year, 1.0% in Year 1 to 0.0% in Year 4 in Jianwu; and Year 1 to Year 4 increases in Koutou from 0.5% to 1.8% and 0.6% to 0.8% in Xiajia (Table 4).

### 3.4. Cost-Effectiveness of the Comprehensive Control Program (Case Villages)

The total costs of the comprehensive control program increased 25× in Year 2 from the baseline control costs (Year 1 = US$10,000 unit cost) and 30x baseline costs in Year 3 in Zhangjia. Increases in costs were also seen in Jianwu, at Year 2 of 1.3× and Year 3 costs of 12.4× baseline. Upon returning to the Standard control program in the case villages in Year 4, the costs returned to pre-intervention levels. In the control villages, the costs remained static during the study period at US$10,000 unit cost (Table 5 and Table 6).

Using the SCI formula described above, SCI in the case village of Zhangjia increased incrementally by 374.89× (Year 2) and 544.82× (Year 3), while SCI increased by 226.65× (Year 2) and 1,149.41× (Year 3) in Jianwu. Further, in Zhangjia the cost effectiveness ratio increased from baseline by 99.68% in Year 2 and 99.98% in Year 3, while the costs in Jianwu increased by 96.67% in Year 3 (Table 7).

Evaluation of the costs associated with the Comprehensive Program for reducing human *S. japonicum* infection by 1% in Zhangjia during Years 2–4, per US$10,000 unit cost, was 0.41, 0.25 and 0.008, and decreased from the Year 1 baseline by 10× in Year 2, Year 3 at 17×, and 483× in Year 4. Decreases were also seen in Jianwu at 0.04, 0.43 and 0.008, respectively; with reductions from the baseline costs in Year 2 of 601×, 59× in Year 3, and Year 4 of 2,738×. In control villages, per year unit costs for the Standard control program was static at US$10,000 unit cost per year. Comparing human infection Comprehensive Program to Standard program costs for Year 2–4 identified Standard program costs to be 0.05, 0.02, and 0.02 (Koutou), and ¬0.02, 0.04, and ¬0.004 (Xiajia) that of the Comprehensive Program intervention costs. At the end of Year 4, the unit cost of the Standard control Program was 2.95 times that of the case village Comprehensive Program.

**Table 4:** The results of snail survey in four years.

		Areas of the snails habitant				Average density of the living snails				Density of the positive snails				Infection rate			
		(ha)				(# counted snails/0.1 m^2^)				(# counted snails/0.1 m^2^)				(% prevalence)			
	Village	Year 1	Year 2	Year 3	Year 4	Year 1	Year 2	Year 3	Year 4	Year 1	Year 2	Year 3	Year 4	Year 1	Year 2	Year 3	Year 4
Cases	Zhangjia	62.53	62.53	62.53	62.53	0.1424	0.0782	0.2035	0.1411	0.0048	0.0009	0.0010	0.0010	3.4	1.1	0.5	0.7
	Jianwu	903.47	903.47	903.47	903.47	0.3081	0.4681	0.3934	0.3593	0.0032	0.0028	0.0004	0.0000	1.0	0.6	0.1	0.0
Control	Koutou	25.40	25.40	25.40	25.40	0.3458	0.4917	1.1365	0.2787	0.0016	0.0036	0.0058	0.0049	0.5	0.7	0.5	1.8
	Xiajia	65.00	65.00	161.81	65.00	0.4836	0.5523	0.6248	1.0535	0.0027	0.0035	0.0019	0.0087	0.6	0.6	0.3	0.8

**Table 5:** The total unit cost of the experimental villages and control villages (Unit: US$ Ten thousand).

	Village	Year 1	Year 2	Year 3	Year 4
Cases villages	Zhangjia	0.48	12.00	14.27	0.48
	Jianwu	1.72	2.15	21.39	0.63
Control villages	Koutou	0.31	0.37	0.62	0.40
	Xiajia	0.60	0.57	0.82	0.60

**Table 6:** Cost of different measures on schistosomiasis control (Unit: US$ Ten thousand).

	Village	Cost, human/bovine + treatment					Cost, snails surveys + elimination					Cost, health education					Cost, comprehensive program				
		Year 1	Year 2	Year 3	Year 4	Total	Year 1	Year 2	Year 3	Year 4	Total	Year 1	Year 2	Year 3	Year 4	Total	Year 1	Year 2	Year 3	Year 4	Total
**Cases**	Zhangjia	0.26	0.31	0.25	0.31	1.13	0.17	0.16	0.15	0.14	0.62	0.05	0.06	0.18	0.03	0.32	0.00	11.47	13.69	0.00	25.16
	Jianwu	0.21	0.22	0.27	0.33	1.03	0.18	0.57	2.56	0.28	3.59	0.05	0.07	0.19	0.02	0.33	1.28	1.29	18.37	0.00	20.94
**Control**	Koutou	0.17	0.19	0.35	0.33	1.04	0.14	0.17	0.18	0.01	0.50	0.00	0.001	0.09	0.06	0.15	0.00	0.00	0.00	0.00	0.00
	Xiajia	0.38	0.42	0.39	0.39	1.58	0.22	0.15	0.41	0.19	0.97	0.00	0.003	0.01	0.005	0.018	0.00	0.00	0.00	0.00	0.00

**Table 7:** Analysis of the comprehensive effectiveness in experimental villages.

Villages		Total cost			Comprehensive impact index			Ratio of	
		(US$ Ten thousand)						cost/index	
	Year 2	Year 3	Year 4	Year 2	Year 3	Year 4	Year 2	Year 3	Year 4
Zhangjia	12.00	14.27	0.48	0.28	105.25	47.58	42.86	0.14	0.01
Jianwu	2.15	21.39	0.63	16.21	52.95	149.58	0.13	0.40	0.004

Evaluation by unit cost of reducing bovine *S. japonicum* infection by 1% in case villages during Years 2–3, was 12.06 and 0.14 in Zhangjia, and Years 2–4 in Jianwu of 42.94, 0.29 and 0.01. There were no bovines in Zhangjia in Year 4. Comparing bovine infection Comprehensive Program to standard program costs for Year 2–4 identified Standard program costs to be 0.015, ¬0.013 and ¬0.06 (Koutou), and 0.04, ¬0.03 and 0.03 (Xiajia) that of the comprehensive program intervention costs. At the end of Year 4, the above indicator of Standard control program costs was 19.3 times than the case village Comprehensive Program (Table 8).

**Table 8:** Cost-effectiveness analysis for four villages.

Village	Reduction in human infection rate (%)			Cost of 1% reduction in human infection rate (US$ Ten thousand)			Reduction in bovine infection rate (%)			Cost of 1% reduction in bovine infection rate (US$ Ten thousand)		
	Year	Year	Year	Year	Year	Year	Year	Year	Year	Year	Year	Year
	4	4	3	4	3	4	4	3	4	3	4	3
Zhangjia	29.40	56.60	55.28	0.41	0.25	0.008	0.00	100.00	0.00	12.06	0.14	—
Jianwu	50.10	48.90	67.23	0.04	0.43	0.008	0.00	75.00	100.00	42.94	0.29	0.01
Koutou	7.69	38.50	20.75	0.05	0.02	0.02	25.00	¬48.22	¬7.05	0.015	¬0.013	¬0.06
Xiajia	¬2.50	27.80	¬119.86	¬0.02	0.03	¬0.004	10.04	¬25.00	20.40	0.04	¬0.03	0.03

### 3.5. Discussion

The present study systematically compared the cost-effectiveness between a comprehensive schistosomiasis control program (Comprehensive Program) and routine control program (Standard program) in four villages of the Poyang Lake region. To our knowledge, this is the first study to assess the cost-effectiveness of a comprehensive control program initiated in China. Our findings indicate that the Comprehensive Program reduced the median prevalence of schistosomiasis 8.5 times below 2003 levels. Further, the cost effectiveness ratio demonstrated that the Comprehensive Program was 170% (Zhangjia) and 922.7% (Jianwu) more cost-effective than the standard intervention. 

Although the initial Year 2 and Year 3 costs associated with Comprehensive Program implementation were higher than the Standard program in the second year and third year in case villages, the cost in the fourth year returned to that in the first year. Further, despite typical year-to-year disease prevalence variability associated with environmental parasitic infections the prevalence of *S. japonicum* infection significantly declined from 11.3% to 1.6% in Zhangjia village and from 6.7% to 0.6% in Jianwu village during the study period. Simultaneously, the infection rate of snails declined from 3.4% to 0.7% in Zhangjia village and there were no infected snails found in Jianwu village during the study period, while the prevalence in control villages remained high. 

Historically, the strategy to control the transmission of *S. japonicum* in China has shifted three times. At the early phase of schistosomiasis control, comprehensive approaches with emphasis on snail elimination were carried out. Since praziquantel was introduced in the 1980s, the strategy shifted to a praziquantel-based integrated “treatment/prophylactic-plus snail elimination” control intervention. This integrated control intervention was implemented in the five Provinces of Shanghai, Fujian, Guangxi, Guangdong and Zhejiang, where transmission interruption was achieved [6]. 

Broadly, Chitsulo and Engels both have identified that successful schistosomiasis control programs should include: (1) national/regional conviction that schistosomiasis is a public health challenge; (2) willingness of local leaders to invest resources to control schistosomiasis; (3) establishment of basic schistosomiasis control and prevention infrastructure [16,17]. In China, the effectiveness of schistosomiasis control is considered to mainly depend on 3 rates: (1) the incidence of acute patient, and associated infection control and reduction; (2) the simultaneous infection rate of humans and livestock; and (3) the infection rate of snails [18]. Livestock, notably bovine, is currently the major source of infection in the lake regions of China. The Comprehensive Program evaluated in this study includes replacement of bovines with mechanization, in addition to the Standard means of control, which focuses solely on elimination of the source of infection. Consequently, as demonstrated by the data from this study, the novel Comprehensive Program approach significantly reduced the prevalence of *S. japonicum* over the Standard methods. Further, the Comprehensive Program strategy not only proved more effective at reducing schistosomiasis prevalence, but also synchronously accelerated local development and economies [19,20].

Sustainability of community disease control efforts requires both, pragmatic effectiveness and economic value. As such, economic evaluation of disease control interventions is increasingly relevant when different control programs are being considered. A generalized static economic framework for investigating the optimal strategy for schistosomiasis chemotherapy was developed [21]. For example, Prescott noted that in sub-Saharan Africa the reduction in costs of praziquantel influenced the ranking of *S. haematobium* infection control measures. Following, a static model and two dynamic models to assess the cost-effectiveness of schistosomiasis control were reviewed [22,23]. In China, a similar assessment was initiated in the early 1990s [24,25]. The economic evaluation method used during this, and subsequent, schistosomiasis control studies is well established [26,27,28,29].These same methods were applied in the present study. While every attempt was made to limit the differences between case and control villages, this evaluation was limited by the variability between village economies and human/animal populations. Additionally, budget limitations and study duration/scale complicated the efforts to minimize confounding and site variability. 

However, WHO has identified a need to effectively evaluate how technological developments impact schistosomiasis transmission and prevalence. Our study is a model of such evaluation. For example in 2004, there were 7 schistosomiasis endemic provinces in China. Starting that same year the Chinese government created a high-priority, focused, national program for schistosomiasis control that applied integrated Comprehensive Programs. Efforts brought to bear the resources of the National Health and Family Planning Commission, State Forestry Administration, Ministry of Agriculture, Ministry of Water Resources, Ministry of Finance, Ministry of Land Resources, and the National Development and Reform Commission. By 2012, those7 endemic provinces were reduced to four that officially reached the stage of “endemic situation control ” and three reached “transmission control”, with the infection rate being less than one percent. The results from this study strongly support the effectiveness of more comprehensive schistosomiasis control interventions. 

## 4. Conclusions

Motivated by remaining challenges associated with continued schistosomiasis endemicity in the lower Yangtze River Basin of China [30,31], a new comprehensive strategy to control and prevent the disease was developed following this study and the effectiveness of these efforts are currently under evaluation [32]. For the first time in China, this study assessed the cost-effectiveness of a Comprehensive Program compared to standard methods and identified the comprehensive approach to be both highly effective at reducing schistosomiasis infection and an excellent return on investment.
